# Long-term calcitonin after thyroidectomy for medullary thyroid cancer in MEN2A

**DOI:** 10.1530/ERC-25-0366

**Published:** 2026-01-19

**Authors:** Marijn L van den Berg, Dirk-Jan van Beek, Medard F M van den Broek, Lutske Lodewijk, Annemarie A Verrijn Stuart, Sheila C E J Terwisscha van Scheltinga, Rachel S van Leeuwaarde, Inne H M Borel Rinkes, Menno R Vriens

**Affiliations:** ^1^ Department of Endocrine Surgical Oncology, University Medical Center Utrecht, Utrecht, The Netherlands; ^2^ Department of Endocrine Oncology, University Medical Center Utrecht, Utrecht, The Netherlands; ^3^ Department of Pediatric Endocrinology, Wilhelmina Children’s Hospital, University Medical Center Utrecht, The Netherlands; ^4^ Department of Pediatric Surgery, Princess Maxima Center for Pediatric Oncology, Utrecht, The Netherlands

**Keywords:** MEN2A, thyroidectomy, calcitonin, medullary thyroid carcinoma, prophylactic thyroidectomy, recurrence

## Abstract

Thyroidectomy is recommended for patients with multiple endocrine neoplasia type 2A (MEN2A). American Thyroid Association 2015 guidelines recommend follow-up of calcitonin values after thyroidectomy. The aim of this study was to determine the natural course of calcitonin levels after total thyroidectomy (TTx) in MEN2A patients. Patients with MEN2A who underwent TTx between 1993 and 2019 and had multiple postoperative calcitonin measurements were retrospectively included from our referral center. Long-term serial calcitonin measurements and clinical outcomes were correlated to the first postoperative calcitonin, histopathology and type of TTx (prophylactic versus non-prophylactic). Fifty-two patients underwent TTx after 1993 at a median age of 10 years (range 0–71). Of these, 23 (44%) had no MTC and 29 (56%) had MTC. The median follow-up time was 12 years (range 3–30). Thirty-eight patients had an ‘undetectable’ first postoperative calcitonin, seven ‘within reference range’ and seven ‘above reference range’. Of the 38 patients with an ‘undetectable’ first postoperative calcitonin, 32 remained ‘undetectable’. All 21 patients without MTC and ‘undetectable’ first postoperative calcitonin remained ‘undetectable’. Of the 17 patients with MTC and ‘undetectable’ first postoperative calcitonin, 11 remained ‘undetectable’ and none developed structural recurrence. Twenty-two of the 25 patients undergoing prophylactic thyroidectomy had repeated ‘undetectable’ measurements. Persistent MTC or structural recurrence occurred in six patients; all had MTC, had a detectable first postoperative calcitonin and underwent non-prophylactic TTx. In conclusion, the long-term serial calcitonin values remain undetectable in the majority of the patients with an undetectable first postoperative calcitonin. Biochemical follow-up for patients without MTC and an undetectable first postoperative calcitonin may not be necessary.

## Introduction

Multiple endocrine neoplasia type 2A (MEN2A) is a rare autosomal dominant syndrome caused by a germline mutation in the *RET* proto-oncogene and is associated with medullary thyroid carcinoma (MTC), pheochromocytoma and primary hyperparathyroidism (1). MTC originates from the parafollicular calcitonin-secreting cells (C-cells) (2) and is the main cause of morbidity and mortality in MEN2A. MTC in MEN2A has a penetrance exceeding 95% and often manifests in childhood, whereas pheochromocytoma and primary hyperparathyroidism carry lifetime risks of 50% and 10–30%, respectively (3). Consequently, pre-symptomatic or timely treatment while minimizing the risks of treatment-related complications is essential (4). Total thyroidectomy (TTx) is the sole preventive or curative approach for MTC in patients with MEN2A. Given the strong genotype–phenotype correlation, since 2001, the timing of operation is based on the specific germline mutation and calcitonin levels (2, 5).

Calcitonin is a sensitive biomarker for MTC and C-cell hyperplasia (CCH) (6). Postoperative calcitonin levels are used for outcome assessment and prognostication. The American Thyroid Association (ATA) 2015 guidelines recommend a calcitonin measurement 3 months postoperatively. If ‘undetectable’ or ‘within reference range’, measurements are recommended biannually in the first year and yearly thereafter (2). This recommendation is based on expert opinion, with limited evidence regarding long-term patient-based calcitonin trends after TTx (2, 4, 6, 7, 8, 9, 10, 11, 12, 13, 14, 15, 16, 17, 18, 19). Apart from postoperative MTC surveillance, patients are recommended to undergo lifelong annual biochemical screening for pheochromocytoma and primary hyperparathyroidism starting from 11 to 16 years depending on the *RET* mutation (2). In case of biochemical abnormalities, localization studies are warranted to identify the hyperfunctioning gland (2). Early surveillance and treatment of MTC and pheochromocytoma have contributed to a significant reduction in or avoidance of MEN2-related morbidity and mortality (20). Nevertheless, the screening program carries ethical and practical issues and is associated with a considerable psychosocial impact (21). Patients with MEN2 encounter psychological challenges associated with the disease and have decreased quality of life (22, 23). Intensive follow-up after TTx may result in unnecessary surveillance for MTC, increased healthcare expenses and heightened patient anxiety.

This study aimed to investigate the natural course of calcitonin levels after TTx in MEN2A patients, to enable evidence-based follow-up. In addition, we aimed to identify factors associated with persistently ‘undetectable’ calcitonin levels, to refine the need for biochemical follow-up in specific subgroups – leading to more tailored surveillance programs for MEN2A patients.

## Patients and methods

### Patients

We retrospectively identified all MEN2 patients diagnosed between 1975 and 2019 in the University Medical Center Utrecht (UMCU)/Wilhelmina Children’s Hospital, a tertiary referral center and MEN2 expertise center in the Netherlands. Patients were included if they had MEN2A and had two or more postoperative calcitonin measurements. All patients were screened for a RET mutation (24) and diagnosed in accordance with the leading guidelines for MEN2 at the time (2, 5, 25). Exclusion criteria were more than 1.5 years between TTx and first postoperative calcitonin measurement and if the calcitonin measurements were analyzed in laboratories other than the UMCU laboratory. All data were collected according to a pre-specified protocol, in line with the Dutch multiple endocrine neoplasia type 1 database (26). Written informed consent for publication was obtained from parents (for patients aged less than 12 years), patients (aged ≥ 16 years) or both (patients aged 12–16 years). The study was approved by the Institutional Review Board of the UMCU.

Thyroid surgery was performed by teams consisting of endocrine surgeons, and for children, a pediatric surgeon worked alongside an endocrine surgeon. All surgeons have extensive experience in thyroid surgery and MEN2A. Surgery timing and extent of neck dissection depended on the guidelines at the time, with all patients discussed in multidisciplinary meetings. Patients were classified into prophylactic versus non-prophylactic thyroidectomy according to the ATA 2015 guidelines, based on the type of codon mutation, preoperative calcitonin and age at TTx (2). A pre-symptomatic MEN2A diagnosis was defined as the absence of clinical symptoms of MTC at the time of diagnosis. The timing of measuring the first postoperative calcitonin level and further follow-up was performed according to the leading guidelines over the study period, approximately 3 months postoperatively (2, 25).

### Pathology

The thyroid glands were formalin-fixed and paraffin-embedded according to standard protocols. Hematoxylin and eosin stains were performed, and slides were subjected to carcinoembryonic antigen and calcitonin immunostaining. The pathologist evaluated slides for normal thyroid tissue, CCH, or MTC. In case of MTC, invasiveness, tumor size, focality, radicality and lymph node metastases were assessed.

### Definition of postoperative structural MTC

Structural MTC was defined as i) confirmed MTC in resection specimen, cytology or biopsy, or ii) radiologically suspicious MTC. Radiologically suspicious MTC was defined as signs suspicious for MTC on consecutive imaging consisting of neck ultrasound, computed tomography, magnetic resonance imaging and functional imaging (^18^F-fluorodeoxyglucose positron emission tomography) assessed by an expert radiologist or a nuclear medicine physician. For patients without MTC in the resection specimen, MTC detected during follow-up was considered as the occurrence of structural MTC. For patients with MTC in the resection specimen, this was defined as structural recurrence. Persistent structural MTC was defined as residual MTC after TTx according to the above-mentioned definition within three months after TTx in combination with persistent high calcitonin levels. All doubtful cases, including patients with radiological findings suspicious for MTC, were discussed in an MEN2A expert panel.

### Outcomes

The primary outcome was the course of postoperative calcitonin measurements. The secondary outcome was the occurrence of structural MTC. Different calcitonin assays were used throughout the study period, including radioimmunoassay, (automatic) immunoluminometric assay, immunoradiometric assay and Immulite Siemens. Calcitonin measurements were classified as ‘undetectable’ – which was defined as measurements below the lower limit of the reference range, ‘within reference range’ and ‘above reference range’ based on the assay cutoff values at that time (Table 1). Calcitonin measurements were also calculated as a factor of the upper limit of normal (ULN) of the reference value.

**Table 1:** Reference range of calcitonin for different calcitonin assays used during the study period.

Period	Assay method	Lower limit Ctn	Upper limit Ctn
Before September 1997	Radioimmunoassay	20 ng/L	400 ng/L
September 1997–September 2000	Immunoluminometric assay	1 ng/L	12 ng/L
September 2000–April 2006	Automatic immunoluminometric assay	1 ng/L	12 ng/L
April 2006–March 2007	Immunoradiometric assay	2 ng/L	12 ng/L
March 2007–March 2018	Immulite Siemens	2 ng/L	12 ng/L
March 2018–now	Immulite Siemens	5 ng/L	10 ng/L

Ctn, calcitonin; ng/L, nanogram per litre.

### Statistical analysis

Continuous variables were reported as median (range), and categorical variables as counts (percentages). Kruskal–Wallis tests were used for comparison of continuous variables, and categorical variables were compared using the chi-squared test or Fisher’s exact test.

Spaghetti plots were used to illustrate the course of individual patient’s postoperative calcitonin. Spaghetti plots started at the first postoperative calcitonin and ended at the last measurement known. Stratified analyses were performed based on first postoperative calcitonin levels (‘undetectable’, ‘within reference range’ or ‘above reference range’), histological findings (normal thyroid tissue or CCH versus MTC) and the type of thyroidectomy according to the 2015 ATA guidelines (prophylactic versus non-prophylactic). Kaplan–Meier curves were plotted, and structural disease-free survival probability estimates were obtained (27). For time-to-event analyses, follow-up started at TTx and ended at the occurrence or recurrence of MTC, last visit or death.

*P*-values <0.05 were considered statistically significant. Analyses were performed using SPSS version 29.0 (IBM Corp, USA), and figures were constructed using GraphPad Prism version 10.0 (GraphPad Software, Inc, USA).

## Results

**Figure 1:**
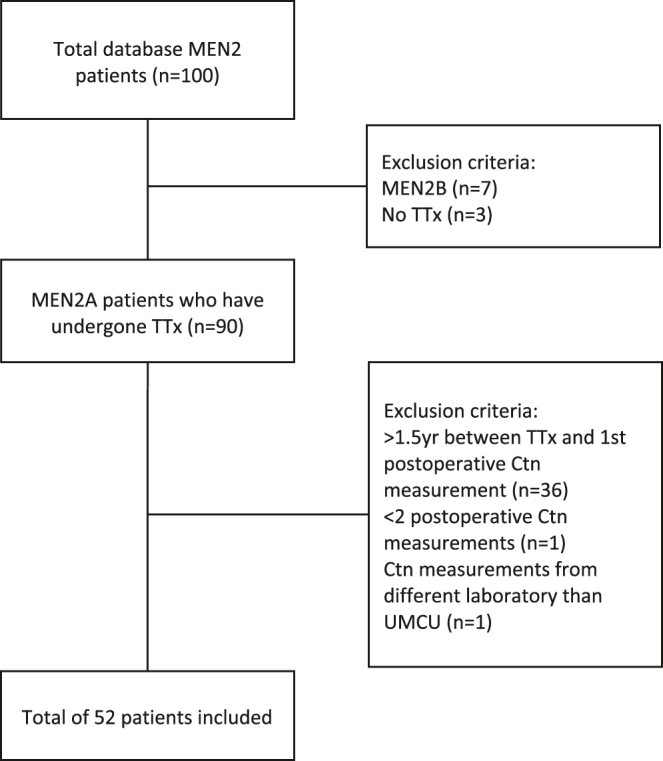
Flowchart of patient inclusion. MEN2, multiple endocrine neoplasia type 2; TTx, thyroidectomy; Ctn, calcitonin; UMCU, University Medical Center Utrecht.

Fifty-two out of 100 MEN2 patients were included (Fig. 1). The baseline characteristics are shown in Table 2. All patients underwent TTx after 1993. The median age at TTx was 10 years (range 0–71). The median age at surgery for patients with pre-symptomatic MEN2A diagnosis who underwent surgery after 2001 is 7 years (range 0–71). Twenty-five patients (48%) underwent a prophylactic TTx according to the ATA 2015 guidelines. Twenty-nine patients (56%) underwent TTx without lymph node dissection (CLND), 17 patients (33%) had TTx with CLND, and 4 (6%) had TTx with CLND and lateral node dissection (LLND). Two patients (4%) had hemithyroidectomy (HT) followed by completion TTx within 3 months, after MTC was found in the HT specimen.

**Table 2:** Baseline characteristics of the total cohort.

	Overall patients (*n* = 52)
Sex ratio (M:F), *n* (%)	26 (50%): 26 (50%)
Age at MEN2A diagnosis (median (range))	9 (0–71)
Codon, *n* (%)	
611	2 (4%)
618	1 (2%)
634	33 (64%)
768	5 (10%)
790	5 (10%)
804	3 (6%)
891	3 (6%)
Patient risk category, *n* (%)	
ATA-H	33 (63%)
ATA-MOD	19 (37%)
Preoperative Ctn levels, *n* (%)	
Normal	12 (23%)
Above upper limit	29 (56%)
Not performed	11 (21%)
Age at surgery in years, *n* (%)	
≤5	20 (39%)
6–10	7 (14%)
11–15	2 (4%)
>15	23 (44%)
Timing of TTx according to ATA guidelines, *n* (%)	
Prophylactic TTx	25 (48%)
Non-prophylactic TTx	27 (52%)
Type of resection, *n* (%)	
HT (followed by completion TTx within 3 months)	2 (4%)
TTx without CLND	29 (56%)
TTx + CLND	17 (33%)
TTX + CLND and unilateral LLND	1 (2%)
TTx + bilateral CLND and LLND	3 (6%)
Histology type, *n* (%)	
Normal thyroid tissue	1 (2%)
C-cell hyperplasia	22 (42%)
MTC	29 (56%)
Focality MTC, *n* (%)	
Unifocal	6 (21%)
Multifocal	18 (62%)
Unknown	5 (17%)
Radicality surgery, *n* (%)	
R0	24 (83%)
R1	2 (7%)
R2	0 (0%)
Unknown	3 (10%)
Categories – MTC size in cm, *n* (%)	
≤1.0	9 (31%)
1.0–2.0	3 (10%)
2.0–4.0	4 (14%)
≥4.0	4 (14%)
Size unknown	9 (31%)
Histology of resected lymph nodes – central compartment, *n* (%)	
Lymph nodes without metastasis	9 (43%)
Lymph node with metastasis	12 (57%)
Histology of resected lymph nodes – lateral compartment, *n* (%)	
Lymph nodes without metastasis	0 (0%)
Lymph node with metastasis	4 (100%)
Time between thyroid surgery and first postoperative calcitonin measurement in months (median (range))	4 (0–14)

MEN2A, multiple endocrine neoplasia; ATA-H, American Thyroid Association high risk; ATA-MOD, American Thyroid Association moderate risk; Ctn, calcitonin; ATA, American Thyroid Association; TTx, thyroidectomy; HT, hemithyroidectomy; CLND, central lymph node dissection; LLND, lateral lymph node dissection; MTC, medullary thyroid cancer; R0, resection microscopically radical; R1, resection macroscopically radical but microscopically not radical; R2, resection micro- and macroscopically not radical.

### Pathology

Pathology showed MTC in 29 patients, CCH in 22 patients and normal thyroid tissue in one patient (Table 2). The histology of the 21 patients who underwent resection of central lymph nodes showed central lymph node metastasis in 12 patients (57%). Two patients (7%), who underwent a TTx with CLND and LLND, had microscopically irradical resections. Both had ‘above reference range’ calcitonin during follow-up.

### First postoperative calcitonin

Thirty-eight patients (73%) had an ‘undetectable’ first postoperative calcitonin, 7 patients (13%) ‘within reference range’ and 7 patients (13%) ‘above reference range’. The majority of patients (6 of 7) with first postoperative calcitonin levels ‘above reference range’ already had preoperative calcitonin levels ‘above reference range’.

The median age at surgery was significantly lower in the ‘undetectable’ calcitonin group (7 years; range 0–71) compared with the ‘within reference range’ group (18 years; range 0–57) and the ‘above reference range’ group (45 years; range 35–57) (*P* = 0.010). In the first postoperative ‘undetectable’ calcitonin group, 17 of the 38 patients had MTC. MTC was observed in five of the seven patients in the ‘within reference range’ group and in all seven patients with ‘above reference range’ calcitonin.

### Long-term course of calcitonin

**Figure 2:**
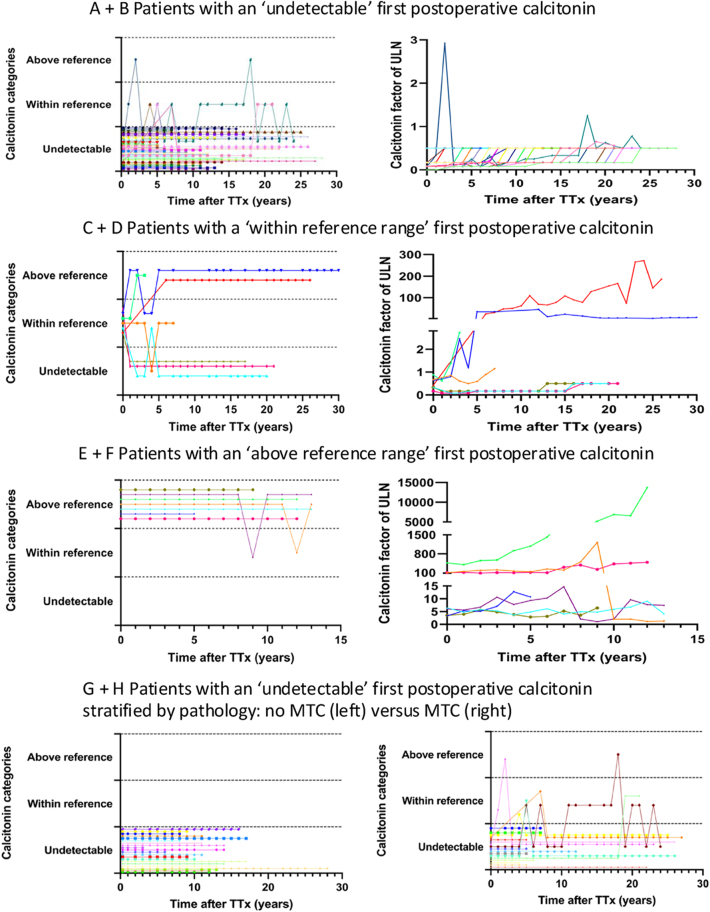
Postoperative calcitonin course during follow-up presented for each individual patient. Ctn, calcitonin; TTx, thyroidectomy; MTC, medullary thyroid cancer; ULN, upper limit of normal. A full color version of this figure is available at https://doi.org/10.1530/ERC-25-0366.

The median follow-up time was 12 years (range 3–30). A total of 926 calcitonin measurements, with a median of 14 (range 3–41) measurements per patient, were available for analysis. Thirty-eight patients had an ‘undetectable’ first postoperative calcitonin level: 32 of the 38 patients remained ‘undetectable’ during follow-up and four of the 38 patients had at least once a ‘within reference range’ calcitonin (Table 3, Fig. 2A). The remaining two patients developed an ‘above reference range’ calcitonin in one single measurement during follow-up, up to a maximum of 3 times above the ULN (Fig. 2B). Subsequent measurements were ‘undetectable’ in one patient and alternated with ‘within reference range’ in the other, both without therapy.

**Table 3:** Characteristics of patients stratified by first measured postoperative calcitonin.

	First measured postoperative calcitonin			*P*-value
	Undetectable (*n* = 38)	Within reference range (*n* = 7)	Above upper limit (*n* = 7)	
Sex ratio (M:F)	19: 19	4: 3	3: 4	0.867
Age at MEN2A diagnosis (median (range))	7 (0–71)	18 (0–57)	45 (35–57)	0.034
ATA risk groups, *n*/*n*				0.406
ATA-H	26/38	4/7	3/7	
ATA-MOD	12/38	3/7	4/7	
Preoperative Ctn levels, *n*/*n*				
Normal	10/38	2/7	0/7	
Above upper limit	22/38	1/7	6/7	
Not performed	6/38	4/7	1/7	
Age at TTx (median (range))	7 (0–71)	14 (4–57)	45 (35–57)	0.010
TTx prophylactic according to the ATA guideline, *n*/*n*				0.018
Prophylactic	22/38	3/7	0/7	
Non-prophylactic	16/38	4/7	7/7	
Type of resection, *n* (%)				
TTx without CND	25/38	4/7	0/7	
TTx + CLND*	13/38	3/7	3/7	
TTx + bilateral CLND and unilateral LLND	0/38	0/7	1/7	
TTx + bilateral CLND and LLND	0/38	0/7	3/7	
Histology type, *n* (%)				0.085
Normal thyroid tissue	1/38	0/7	0/7	
C-cell hyperplasia	20/38	2/7	0/7	
MTC	17/38	5/7	7/7	
Radicality surgery, *n* (%)				
R0	17/38	2/5	5/7	
R1	0/38	0/5	2/7	
R2	0/38	0/5	0/7	
Unknown	21/38	3/5	0/7	
Categories – MTC size in cm, *n* (%)				
≤1.0	7/38	1/5	1/7	
1.0–2.0	1/38	0/5	2/7	
2.0–4.0	2/38	1/5	1/7	
≥4.0	1/38	0/5	3/7	
Size unknown	6/38	3/5	0/7	
Time of FU in years (median (range))	12 (3–28)	17 (3–30)	12 (5–13)	0.378
Number of Ctn measurements, median (range)	11 (3–41)	24 (5–40)	28 (14–41)	0.011
Ctn during FU, *n* (%)				<0.001
Undetectable	32/38	2/7	0/7	
Within reference range	4/38	2/7	0/7	
Above reference range	2/38	3/7	7/7	

MEN2A, multiple endocrine neoplasia; ATA-H, American Thyroid Association high risk; ATA-MOD, American Thyroid Association moderate risk; Ctn, calcitonin; ATA, American Thyroid Association; TTx, thyroidectomy; HT, hemithyroidectomy; CLND, central lymph node dissection; LLND, lateral lymph node dissection; MTC, medullary thyroid cancer; R0, resection microscopically radical; R1, resection macroscopically radical but microscopically not radical; R2, resection micro- and macroscopically not radical; FU follow-up.

* Two patients underwent a hemithyroidectomy followed by completion TTx + CLND within three months.

Seven patients (14%) had a ‘within reference range’ first postoperative calcitonin; two of the seven patients had an ‘undetectable’ calcitonin during follow-up, likely due to the first postoperative measurement being within 1.5 months after TTx. Two of the seven patients remained ‘within reference range’, while three developed ‘above reference range’ calcitonin during follow-up, with two increasing to 90–300 times above the ULN (Table 3, Fig. 2C and D).

Furthermore, all seven patients with ‘above reference range’ first postoperative calcitonin levels remained ‘above reference range’ (Table 3, Fig. 2E and F); two patients showed a decrease in calcitonin during follow-up due to additional surgical and targeted therapy.

### Postoperative calcitonin levels stratified by pathology results

All 21 patients with an ‘undetectable’ first postoperative calcitonin and no MTC had ‘undetectable’ levels in a total of 265 calcitonin measurements (median of 11 measurements per patient) during a median follow-up of 12 years (range 3–28) (Table 3, Fig. 2G).

Of the 17 patients with an ‘undetectable’ first postoperative calcitonin and MTC, 11 did not develop any ‘within reference range’ calcitonin during a median follow-up of 20 years (range 4–27). The remaining six patients developed at least one calcitonin value ‘within reference range’, but only two of them had a single calcitonin level ‘above reference range’ during follow-up (Fig. 2H).

### Postoperative calcitonin levels stratified by prophylactic and non-prophylactic TTx

**Figure 3:**
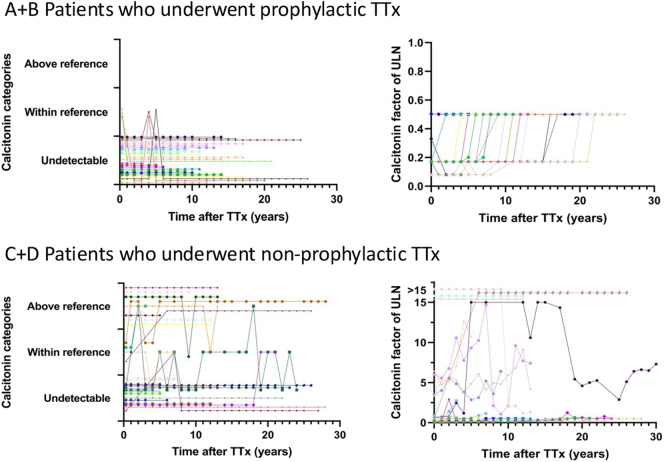
Postoperative calcitonin course during follow-up stratified by prophylactic and non-prophylactic TTx presented for each individual patient. Ctn, calcitonin; TTx, thyroidectomy; MTC, medullary thyroid cancer; ULN, upper limit of normal. A full color version of this figure is available at https://doi.org/10.1530/ERC-25-0366.

Twenty-two of the 25 patients who underwent prophylactic TTx according to the ATA 2015 guidelines had an ‘undetectable’ first postoperative calcitonin. The remaining three had initial calcitonin levels ‘within reference range’, which decreased to ‘undetectable’ without additional treatment. During follow-up, three patients developed a single ‘within reference range’ measurement and the calcitonin of the other 22 patients remained ‘undetectable’ (Fig. 3A and B).

Of the 27 patients who underwent non-prophylactic TTx, 16 had ‘undetectable’, four had ‘within reference range’ and seven had ‘above reference range’ first postoperative calcitonin levels. Calcitonin remained ‘undetectable’ in 12 of 16 patients, and the other four had intermittent ‘within reference range’ levels, which spontaneously decreased to ‘undetectable’ in three patients. Among the four with an initial ‘within reference range’ calcitonin, three had multiple ‘above reference range’ measurements. All seven patients with an initial ‘above reference range’ calcitonin had repeated ‘above reference range’ measurements.

### Structural MTC during follow-up

**Figure 4:**
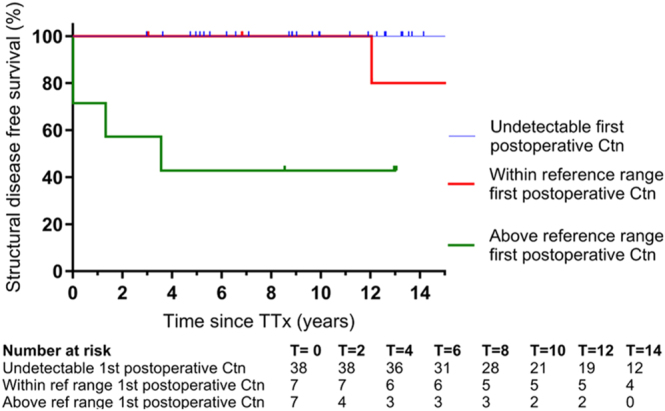
Structural disease-free survival of MEN2A patients after thyroidectomy stratified by first postoperative calcitonin categories. Ctn, calcitonin; TTx, thyroidectomy. A full color version of this figure is available at https://doi.org/10.1530/ERC-25-0366.

No patients with normal thyroid tissue or CCH developed structural MTC during a median follow-up of 12 (3–28) years (Table 4, Fig. 4). In patients with MTC and an ‘undetectable’ first measured postoperative calcitonin, no structural recurrence was observed during a median follow-up time of 20 (4–27) years (Table 4). Structural recurrence occurred in two of the four patients with MTC and a first postoperative ‘within reference range’ calcitonin after 12 and 17 years after TTx (Table 4, Fig. 4). Two of the seven patients with MTC and lymph node metastasis had persistent disease, and another two developed structural recurrence. Structural recurrence occurred only in patients who underwent non-prophylactic TTx.

**Table 4:** Overview of follow-up of calcitonin and incidence of structural MTC stratified by pathology and first postoperative calcitonin levels.

Pathology	First Ctn postop	FU* (range)	Ctn category during FU			Structural MTC			
			UND *n*/*n*	WRR *n*/*n*	ARR *n*/*n*	No RR *n*/*n*	RR *n*/*n*	PD *n*/*n*	TTR* (range)
No MTC	UND	12 (3–28)	21/21	0/21	0/21	21/21	0/21	0/21	-
	WRR	19 (17–20)	1/2	1/2	0/2	2/2	0/2	0/2	-
	ARR	-	0/0	0/0	0/0	0/0	0/0	0/0	-
MTC + no LNM	UND	20 (4–27)	7/13	4/13	2/13	13/13	0/13	0/13	-
	WRR	24 (7–30)	1/4	1/4	2/4	2/4	2/4	0/4	15 (12–17)
	ARR	-	0/0	0/0	0/0	0/0	0/0	0/0	-
MTC + LNM	UND	5 (5–7)	4/4	0/4	0/4	4/4	0/0	0/4	-
	WRR	3 (3)	0/1	0/1	1/1	1/1	0/1	0/1	-
	ARR	12 (5–13)	0/7	0/7	7/7	3/7	2/7	2/7	2 (1–4)

Ctn, calcitonin; FU, follow-up; ref, reference; MTC, medullary thyroid cancer; LN, lymph node.

* Median time in years.

## Discussion

In this single-center cohort study, we investigated the long-term calcitonin levels of patients in patients with MEN2A who underwent TTx. Among patients with an ‘undetectable’ first postoperative calcitonin, 32/38 patients maintained undetectable levels, and no structural occurrence or recurrence of MTC was observed in this group. In particular, all 21 patients without MTC and an ‘undetectable’ first postoperative calcitonin remained ‘undetectable’ during a median follow-up time of 12 years. The majority of the patients (11 of 17) with an ‘undetectable’ first postoperative calcitonin and MTC did not develop any calcitonin measurement ‘within reference range’ or ‘above reference range’ during follow-up, nor did they experience structural recurrence of MTC. Twenty-two of the 25 patients undergoing prophylactic thyroidectomy had repeated ‘undetectable’ measurements. These findings suggest that the current follow-up regimen may be too intensive for a substantial number of patients.

Several studies have investigated long-term outcomes after TTx in MEN2. Rohmer *et al.* and Machens *et al.* found no ‘above reference range’ calcitonin in 140 patients without MTC and in all 167 patients with prophylactic TTx, respectively (8, 9). In a multicenter study of 67 MEN2A children with a median follow-up of 104 months, calcitonin remained ‘undetectable’ in 27 patients, ‘within reference range’ in 19 patients and ‘above reference range’ at least once in 21 patients (12). Skinner *et al.* observed that 44 of the 50 (88%) MEN2 patients who were operated on at or before the age of 19 had calcitonin that stayed ‘undetectable’ or ‘within reference range’ (13). Febrero *et al.* reported that only two of the 50 MEN2A patients developed an ‘above reference’ calcitonin after a median follow-up of 16 years (14). These studies indicate that prophylactic or early TTx in MEN2 patients typically results in ‘undetectable’ or ‘within reference range’ calcitonin levels. Advances in MEN2-related MTC management have shifted from treating clinically evident disease to risk-adapted, codon-based prophylactic TTx, substantially improving outcomes and questioning the necessity of routine long-term follow-up (24).

After the complete removal of thyroid tissue in MEN2, calcitonin levels should be ‘undetectable’ (9, 10, 11). Current guidelines recommend lifelong annual calcitonin measurements (2). The ATA guidelines group patients with ‘undetectable’ and ‘within reference range’ calcitonin together, but our study shows that among the ‘undetectable’ group, only 6 out of 38 patients developed a ‘within reference’ or ‘above reference range’ calcitonin during follow-up, compared to 5 out of 7 patients with a ‘within reference’ first postoperative calcitonin. Based on our findings, these two groups should be regarded, counseled and followed as distinct entities. Future studies should also incorporate other factors, such as histopathological characteristics, the affected codon and the timing of TTx.

Based on our findings, we propose a more individualized calcitonin follow-up regimen. This study shows that in 52 patients, 926 calcitonin samples were drawn, and only six patients had structural recurrence. Early postoperative risk stratification could improve postoperative counseling of patients and reduce uncertainty and anxiety (20, 21). In addition, reducing follow-up would cut laboratory tests and lower healthcare costs. For patients without MTC and an ‘undetectable’ first postoperative calcitonin, follow-up appears unnecessary, as none of these patients developed a ‘within reference range’ or ‘above reference range’ postoperative calcitonin or structural MTC. We suggest discontinuing the MTC surveillance regimen for these patients. While patients still require follow-up for thyroid hormone supplementation after TTx, pheochromocytoma and primary hyperparathyroidism screening, eliminating calcitonin follow-up would remove the need for testing in a referral center.

Patients with MTC and an ‘undetectable’ first postoperative calcitonin did not develop structural recurrence during a median follow-up of 20 years (range 4–27) with calcitonin remaining undetectable in 11/17 of cases. The remaining six patients developed at least one ‘within reference range’ calcitonin measurement, of whom two had a single ‘above reference range’ calcitonin measurement, followed by alternating ‘within reference range’ and ‘undetectable’ levels in one patient, and ‘undetectable’ levels in the other. Postoperative surveillance for these patients can likely be shortened, or the intensity can be reduced.

This study is, to our knowledge, unique in the way we studied the postoperative calcitonin course in relation to the pathological outcomes and the first postoperative calcitonin value (2, 4, 6, 7, 8, 9, 10, 11, 12, 13, 14, 15, 16, 17, 18, 19). Every postoperative calcitonin measurement for each patient was analyzed using spaghetti plots. The follow-up period was relatively long, as loss to follow-up was minimized by the patient adherence to lifelong follow-up in our center. Considering the rarity of MEN2, the number of patients included in our study is substantial. Although data collection was performed retrospectively, it followed a pre-specified protocol without interpretation by data collectors (26). Limitations include its retrospective design with data dating back to 1990, which may not fully reflect current MEN2A protocols. Multiple calcitonin assays were used during the study period, with varying reference ranges and sensitivities. A less sensitive calcitonin assay (with potential cross-reaction) was used from 2000 to 2006, which could have led to false-detectable calcitonin levels. As a result, the number of patients with ‘undetectable’ calcitonin during follow-up may have been slightly underestimated. In addition, the first postoperative calcitonin measurement should ideally be performed after three months (2), considering the half-life of calcitonin, to avoid false-detectable calcitonin levels after thyroidectomy (28). Thirty-six patients who had their first postoperative calcitonin measurement more than 1.5 years after thyroidectomy were not included; the uncertainty regarding calcitonin levels during this extended interval prompted their exclusion to ensure a homogeneous cohort. Multivariable analyses were not possible due to the low number of patients in specific subgroups. Although the follow-up was long, it was not lifelong, which limits the ability to rule out very late occurrences or recurrence of MTC. Finally, because this study was performed in an expert center, our results must be validated in other MEN2A cohorts to assess the generalizability of our outcomes.

In conclusion, in this study, we investigated which patients developed biochemical or structural disease after TTx in MEN2A, with the aim of individualizing follow-up. Our results suggest that long-term biochemical follow-up is unnecessary for patients without MTC and an ‘undetectable’ first postoperative calcitonin. The calcitonin follow-up for patients with MTC and an ‘undetectable’ first postoperative calcitonin can likely be reduced or shortened.

## Declaration of interest

The authors declare that there is no conflict of interest that could be perceived as prejudicing the impartiality of the work reported.

## Funding

This work received no specific grant from any funding agency in the public, commercial or not-for-profit sectors.

## Author contribution statement

MLvdB and DJvB contributed substantially to the conception and design of the study. MLvdB was responsible for data acquisition, performed the statistical analyses, and led the interpretation of the data. DJvB contributed to the statistical analyses and interpretation. MFMvdB and RSvL were founding contributors to the database and provided expert input, particularly regarding MEN2A. LL , AAVS, SCEJTvS, IHMBR, and MRV contributed to the interpretation of the data and provided clinical expertise. All authors were involved in drafting the manuscript or revising it critically for important intellectual content. All authors approved the final version of the manuscript.

## Data availability

The data that support the findings of this study are available from the corresponding author upon reasonable request.
